# Histologic effects of mandibular protrusion splints in antigen-induced TMJ arthritis in rabbits

**DOI:** 10.1186/s12969-017-0158-0

**Published:** 2017-04-13

**Authors:** Julia von Bremen, Kernt Köhler, Krystyna Siudak, Daniel Zahner, Sabine Ruf

**Affiliations:** 1 Department of Orthodontics, University of Giessen, Schlangenzahl 14, 35392 Giessen, Germany; 2 Institute of Veterinary Pathology, University of Giessen, Giessen, Germany; 3 Animal laboratories, University of Giessen, Giessen, Germany

**Keywords:** JIA, Arthritis, TMJ morphology, Functional mandibular advancement

## Abstract

**Background:**

Although it is common clinical practice to treat children with Juvenile Idiopathic Arthritis (JIA) with functional appliances, the scientific evidence for this is limited. The aim of this study was to study the histologic effects of mandibular protrusion splints in temporomandibular joint (TMJ) arthritis in rabbits.

**Methods:**

Twenty-eight ten-week old New Zealand white rabbits were randomly divided into four groups: AO (TMJ arthritis, no splint), AS (TMJ arthritis, mandibular splint advancement), OS (no arthritis, mandibular splint advancement) and OO (no arthritis, no splint). TMJ arthritis was induced in the groups AO and AS; 1 week later mandibular protrusion splints were placed on the upper incisors of the AS and OS animals. After 60 days the animals were sacrificed and a semiquantitative histologic evaluation of each TMJ was carried out to analyze the amount of inflammation and bone modeling.

**Results:**

AO and AS animals had a higher inflammation score (AO = 1.3; AS = 1.8) than the non-arthritis groups (OO = 0.6; OS = 0.4). Whereas in the untreated control (OO) the amount of apposition and resorption was almost in balance (+1), OS animals displayed significantly more apposition (+9) and AO animals significantly more resorption (−3) than the untreated control. Arthritis animals with protrusion appliances (AS), however, had remarkably more bone apposition (+3) than resorption, indicating a similar bony reaction as in healthy animals, although reduced in extent.

**Conclusions:**

Mandibular advancement in rabbits with TMJ arthritis is possible without detrimental histologic reactions and appears to partially compensate for the bone loss seen in rabbits with TMJ arthritis but without protrusion splints.

## Background

Juvenile idiopathic arthritis (JIA) is one of the most common chronic diseases in childhood, with a reported prevalence of 1 in 1000 children [1]. The prevalence of clinically detectable temporomandibular joint (TMJ) involvement varies between 38 and 72%, depending on the diagnostic method used and the JIA-subtype examined [2–8]. This TMJ inflammation may cause significant limitations in sagittal and vertical mandibular growth, conditionally resulting in severe micrognathia and anterior open bites with marked esthetic and functional restrictions [2, 3, 9–19].

Overall, the evidence on orthodontic treatment principles for JIA-children with TMJ involvement is low. However, there is limited evidence that dentofacial orthopedic treatment using functional appliances can improve mandibular retrognathia and reduce pain in adolescent JIA-patients [20]. It has also been demonstrated that in cases with unilateral TMJ involvement, resulting in an asymmetrical mandibular growth, functional appliance treatment can reduce these asymmetries [21]. Thus, it appears to be clinically possible to enhance growth in JIA children with TMJ involvement to a certain extent and partially compensate for the growth limitations caused by the inflammatory process [22–24].

Nevertheless, some authors advise not to “strain” the TMJ, as would be the case during mandibular advancement procedures, because they fear accelerated condylar destruction of the TMJ as a result of an increased bone turnover rate [25]. Considering the results of basic cell research, however, these fears seem unnecessary, since numerous in vitro studies have shown an anti-inflammatory effect of tensile strain on inflamed chondrocytes [26–29]. Furthermore, condyles have an exceptional potential for regeneration and remodelling, to an extent that even in growing rats, where the condyles had been experimentally resected, growth could be enhanced through mandibular advancement [30].

For healthy individuals, it is well known that treatment with functional appliances induces bone modelling in the TMJ [31, 32]. Furthermore, it has been shown that patients treated with a Herbst appliance displayed a certain amount of regeneration of arthrotic TMJ-lesions [33, 34]. Therefore, it was assumed that also children with an arthritic TMJ involvement could benefit from functional mandibular advancement with a fixed functional appliance. Since, however, in JIA children many cofactors can influence the reaction (disease duration, general medication, degree of TMJ involvement, age, subtype etc.), making it extremely difficult, if not impossible, to get a patient cohort with a comparable situation, it was decided to analyse the treatment effects in an animal model.

The aim of this animal study was to analyse the histologic effects of mandibular protrusion appliances in rabbits with antigen induced arthritis of the temporomandibular joint. The null hypothesis was that there would be no histologic differences concerning inflammation or bone remodelling in the TMJs of rabbits with an antigen induced arthritis between animals treated with or without mandibular protrusion appliances.

## Methods

Twenty-eight ten-week old New Zealand white rabbits (*Oryctolagus cuniculus*) were housed at the central laboratory animal facilities of the University of Giessen, Germany and had constant access to food and water. To ensure that the protrusion appliances would not hinder the animals from eating, the food (dried pellets) was moistened for all animals. Animal welfare was supervised daily by weight control as well as evaluation of food and water intake. All procedures were approved by the government ethical committee for animal welfare in Giessen, Germany (71/2008).

**Fig. 1:**
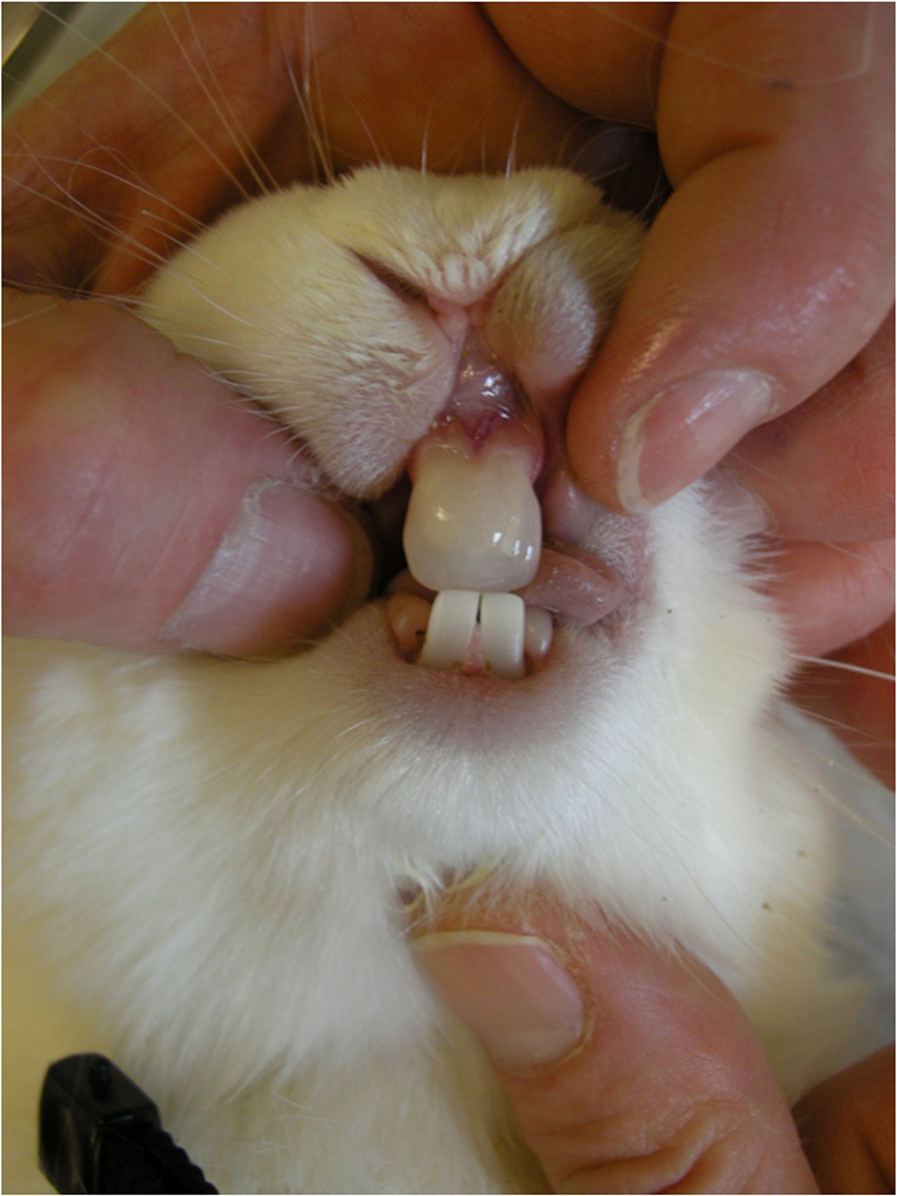
Bonded mandibular protrusion appliance on upper incisors of a rabbit

Upon arrival (age 8 weeks), the subjects were randomly divided into one of four groups: group A0 (TMJ arthritis, no appliance; 7 animals), group AS (TMJ arthritis, mandibular splint advancement; 7 animals), group 0S (no arthritis, mandibular splint advancement; 7 animals), and group 00 (control group: no arthritis, no appliance; 7 animals) (Table 1). According to the method described by Kapila et al. [35] all animals were presensitized (age 10 weeks) and sensitized (age 12 weeks) with ovalbumin (see supplemental file). After confirmation of sensitation, the animals of groups A0 and AS received a bilateral intraarticular ovalbumin injection to induce TMJ arthritis (age 13 weeks). One week later (age 14 weeks) mandibular protrusion appliances were placed on the upper incisors of the AS and 0S animals, advancing their mandible to an incisal edge-to-edge position (Fig. 1). Intraarticular injections and appliance placement were carried out under general anesthesia (Ketamin 0.25 mg/kg and Medetomidin 35 mg/kg). After 60 days the animals were sacrificed and the TMJs were retrieved. Decalcification was performed with EDTA (20%) at 37 °C for at least 14 days. Afterwards they were embedded automatically in paraffin wax according to a routine protocol (Sakura VIP 5 jr. tissue processor). The sectioning at 4 μm was performed using a microtome (Leica RM2255), the staining with hematoxilin-eosine and Periodic Acid Schiff (PAS) method. All histologic evaluations were performed independently by two very experienced veterinary pathologists (KK and KS), who were blinded for both arthritis and protrusion splint wear. Despite the intent to perform a quantitative analysis, we had to realize that it was impossible to get identical sections of the joints which allowed for only a semiquantitative analysis of both inflammation and bone modelling. Therefore, the degree of inflammation was scored from 0 (none) to 3 (massive) according to the amount of plasma cells, lymphocytes and synovial proliferation as well as blood vessels or lymphatic tissue (as an indicator for chronic inflammation) or heterophilic granulocytes (as an indicator for pus; acute inflammation) (Table 2). The semiquantitative analysis for bone modelling assessed both resorption according to the amount of osteoclasts (0 = none to −3 = massive) and bone formation according to the amount of osteoblasts and newly formed osteoid (0 = none, +3 = massive) for each joint. The total score for apposition and resorption for each joint was then calculated, with values above 0 indicating that bone apposition prevailed, whereas values below 0 indicated a dominance of bone resorption (Table 2). This was performed for each joint. The statistical evaluation, however, was performed on the animal level, which means, that only that joint side of each rabbit was evaluated, which had the most severe signs of inflammation and the most catabolic bone situation. After the independent analysis by both pathologists the cases of disagreement were solved by consensus.

**Table 1:** Randomized distribution of the 28 New Zealand White rabbits into the different experimental groups (A0, AS, 0S, 00)

	Arthritis	No arthritis
Protrusion splint	AS (*n* = 7)	A0 (*n* = 7)
No protrusion splint	0S (*n* = 7)	00 (*n* = 7)

**Table 2:** Criteria for the semiquantitative scoring of inflammation and bone modelling

	Analyzed factors
	Inflammation
Score	•plasma cells •lymphocytes •heterophilic granulozytes •lymphatic tissue synovial proliferation
0	none
1	slightly present
2	moderately present
3	severely present
	Bone modelling
Score	•osteoblasts •osteoclasts •osteoid formation •reversal lines
−1	slightly more resorption than apposition (catabolic situation)
0	amount of resorption equals apposition (balanced situation)
+1	slightly more apposition than resorption (anabolic situation)
+2	moderately more apposition than resorption (anabolic situation)

## Statistical analysis

Statistical calculations were done with SPSS Statistics 22 (SPSS Inc. an IBM Company, Chicago, IL). All tests were performed two sided with a significance level of 5%. An alpha adjustment for multiple testing was not applied, and the results were interpreted accordingly as being explorative. Ordinally scaled histological values were displayed in absolute and percent frequencies. Groups or sides were compared regarding these values in contingency tables and tested for dependence with chi-square trend test.

## Results

Unfortunately, two animals of the A0 group were lost during induction of the TMJ arthritis: the first due to respiratory arrest under general anesthesia, the second due to an anaphylactic shock. All others completed the trial without complications with a normal weight development.

### Inflammation

**Fig. 2:**
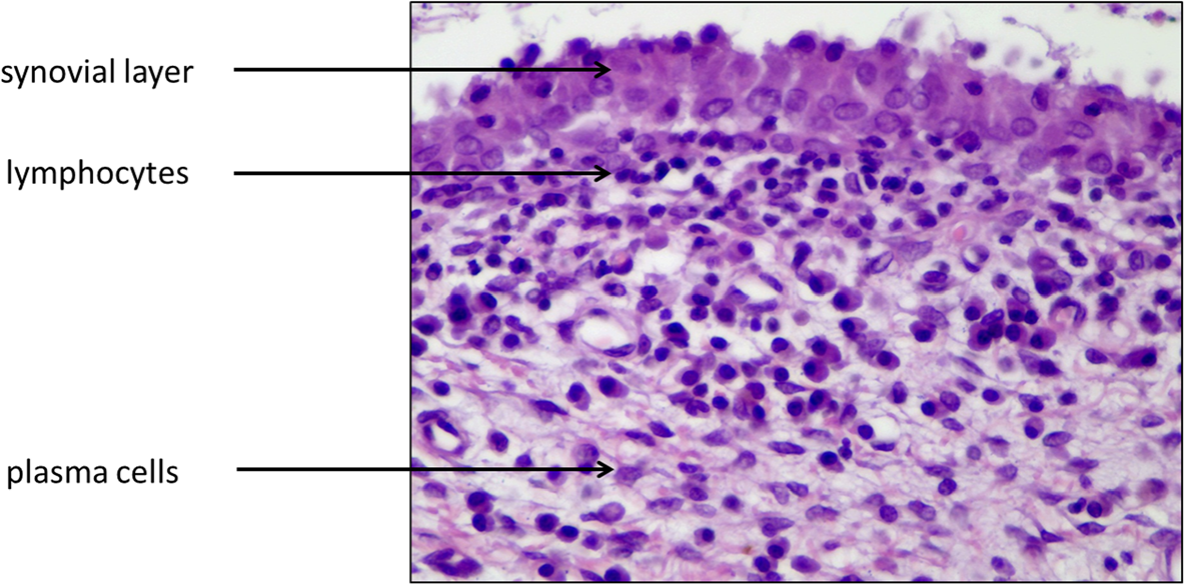
Example of a typical chronic inflammation of the condylar synovial layer in a rabbit TMJ (40x)

**Fig. 3:**
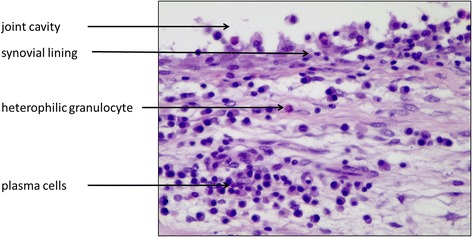
Example of a typical acute inflammation of the condylar synovial layer in a rabbit TMJ (40x)

While the inflammation score for the groups without arthritis lay between “none” and “slight”, the score for the two arthritis groups lay between “slight” and “moderate”. On average the 0S-animals had slightly lower values (0.4) than the 00 group (0.6), whereas in comparison of the two arthritis groups higher values were found for the animals with protrusion appliances (AS = 1.8) than for those without mandibular protrusion (A0 = 1.3) (Table 3). In all groups (including the untreated control), areas with a synovial proliferation or an accumulation of plasma cells could be identified (Fig. 2), the arthritis groups additionally often displayed an accumulation of heterophilic granulocytes (Fig. 3).

**Table 3:** Inflammation scores (absolute) and indexes (relative) for the four experimental groups (00, 0S, A0, AS): 0 = none, 1 = slight, 2 = moderate, 3 = severe

	Analyzable joints (*n*)	Inflammation score (absolute)	Inflammation index (relative)
00	13	8	0.6
0S	14	5	0.4
A0	10	13	1.3
AS	13	24	1.8

**Fig. 4:**
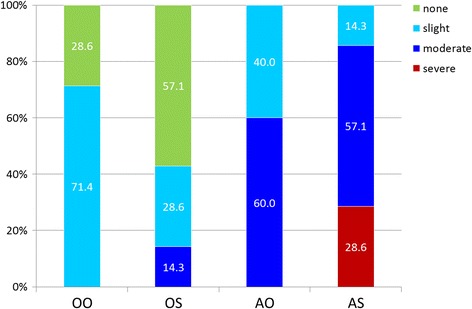
Inflammation scores in the four experimental groups. Comparison of the more severely affected joint side of each animal

When evaluating exclusively the more severely affected TMJ side of each animal, it became clear that most animals of the 00 and 0S groups did not have any or only slight signs of inflammation, whereas 40% of the A0 and only 14.3% of the AS animals fell into these categories. Correspondingly, 85.7% of the animals in the AS group showed a moderate to severe inflammation on their more affected joint side. For the A0 animals this was the case in 60%, for the 0S group in 14.3%. The difference between the groups 00 and A0 or AS was statistically significant (00-A0: *p* = 0.027; 00-AS: *p* = 0.024). An intergroup difference between A0 and AS animals could not be verified (*p* = 0.221) (Fig. 4).

### Bone modelling

**Fig. 5:**
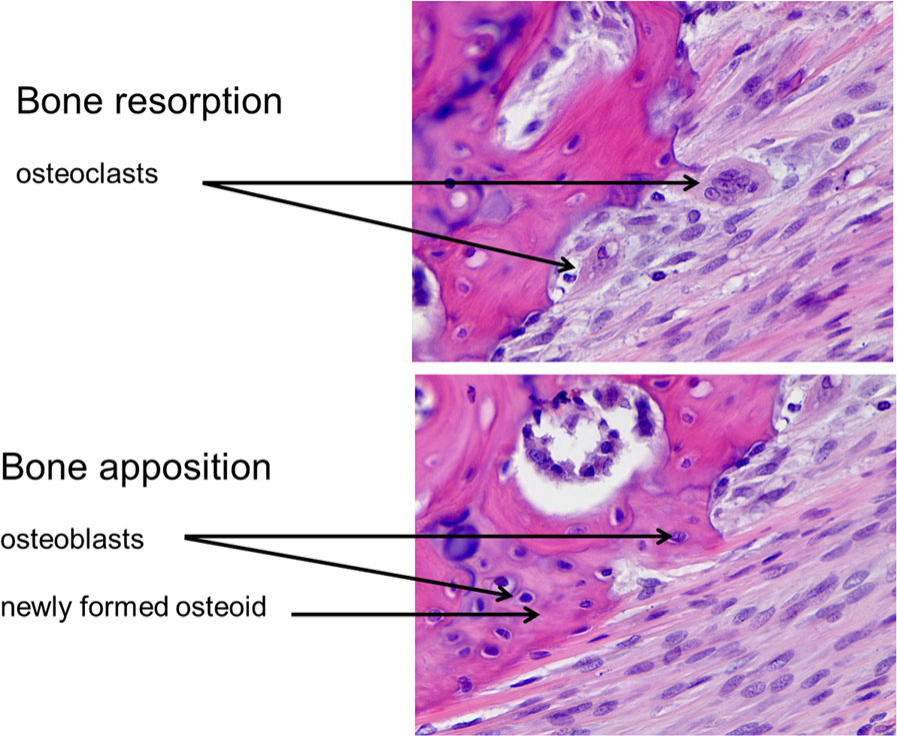
Example of bone modelling in the rabbit TMJ with areas of predominant bone resorption (*top*) and apposition (*bottom*)

**Fig. 6:**
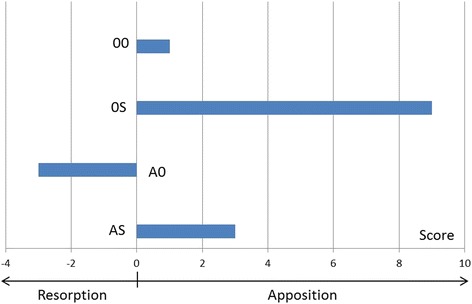
Relation of anabolic and catabolic bone remodelling processes for the different experimental groups

Since all subjects were young, growing rabbits, a certain amount of bone apposition and resorption was found in every TMJ (Fig. 5). When semiquantitatively relating the amount of bone apposition (+) to the amount of resorption (−) for each animal and each TMJ side (Table 2), animals of the untreated control (00) exhibited an almost balanced situation (+1). 0S animals had significantly more apposition than resorption (+9) whereas in the A0 group bone resorption prevailed (−3). If arthritis animals were treated with protrusion appliances (AS), however, they had a similar anabolic bone remodelling situation as the 0S-group (+3), although reduced in extent (Fig. 6).

**Fig. 7:**
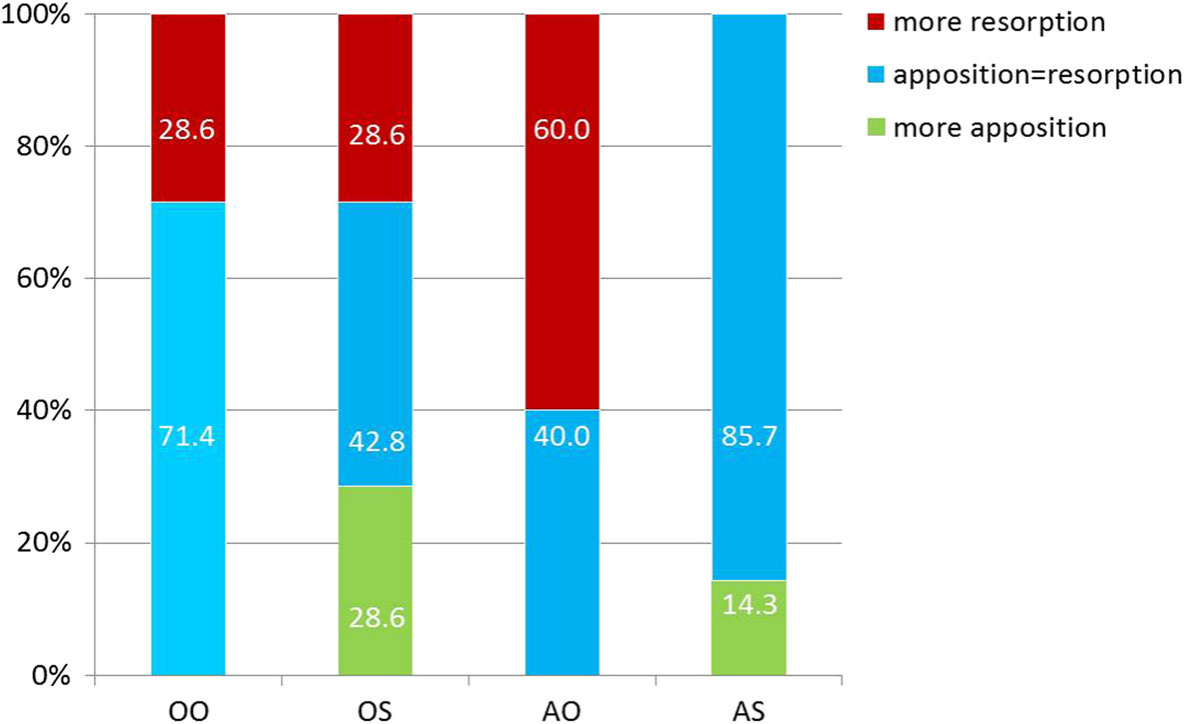
Relation of bone apposition to resorption in the four experimental groups. Comparison of the less anabolic joint of each animal

**Fig. 8:**
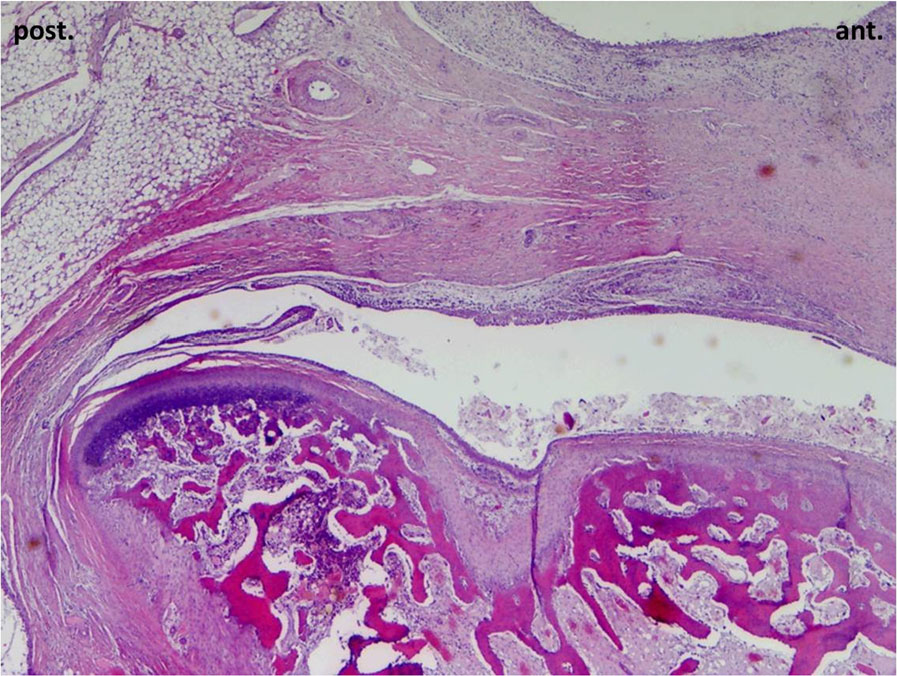
Overview (2x) of a rabbit TMJ with the typical location of a defect of the condylar chondrocyte layer and bone. The posterior condylar region was not affected by degenerative processes in the present sample, but often presented a hyperplasia of the chondrocyte layer (preliminary stage to new bone apposition)

The TMJ side with the most unfavourable bone remodelling situation (much resorption, little apposition) was evaluated statistically. Here, 71.4% of the untreated control (00) has a balanced relation of apposition to resorption. In the 0S group a balanced situation was observed in 42.8% of the joints, whereas 28.6% had more apposition and 28.6% more resorption. For the arthritis animals without mandibular advancement (A0), the majority (60%) had more bone resorption, while 40% had a balanced situation. The AS group, on the contrary, displayed a balanced situation in almost all joints (85.7%). In one animal (14.3%) even more apposition than resorption was found, despite the fact that only the joint with the most “destructive” bone remodelling situation was evaluated. This difference was statistically significant (A0-AS: *p* = 0.035) (Fig. 7). Interestingly, if condylar abnormalities (thinning of chondrocyte layer/bony defects) were found, they were always located in the anterior cranial region of the condyle, whereas the posterior region was not affected (Fig. 8).

## Discussion

### Limitation of the study

To our knowledge, no validated method for a quantitative analysis of histologic changes in the rabbit TMJ has been described, although other authors have performed such an evaluation using varying methods in rabbits and even rats [36–38]. Despite the fact that two very experienced veterinary pathologists performed the present sample preparation (KK) and histological evaluation (KK and KS), it was not possible to obtain the exact same sectional plane in all samples, primarily due to interindividual morphologic differences. Thus, although originally planned, we eventually refrained from undertaking a quantitative analysis. Concerning the reliability of the alternative semiquantitative measurements is was found that both pathologists scored the inflammation and bone resorption very similarly whereas the amount of new bone formation was the main object of discussion. Here in 11 of the 52 samples an initial disagreement concerning the amount of bone apposition was evident, which, however, was agreed upon without problems. Furthermore, it has to be remarked that retrospectively it would have been nice to have had pre- and posttreatment CBCTs to document mandibular growth. Unfortunately this idea did not come to our mind before conducting the trial so that this data is not available. An expected complication, the overgrowth of the incisors, was not observed. It appeared as if the lower incisors showed a more or less normal wear at the end of the observation period, the protrusion appliances were still in place in all animals and the upper incisors were of normal length.

### Inflammation

As expected, the inflammatory markers were higher in the arthritis groups (AS and A0) than in the non arthritis groups (0S and 00). Nevertheless, it has to be remarked that the untreated control group also exhibited a certain amount of inflammatory cells. This might appear surprising, but due to the fact that only growing animals, who constantly exhibit a certain amount of bone remodelling, were analysed, the presence of inflammatory cells is a physiological circumstance. When comparing 00 to 0S animals, slightly less inflammatory markers were present in the group with protrusion appliances. Comparing A0 to AS animals, however, the group with protrusion appliances exhibited more signs of inflammation. This relative increase of inflammation is assumingly an addition of the TMJ arthritis to the reaction observed during mandibular advancement. It has been observed that also systemically healthy dentofacial orthopedic patients (Herbst appliance and Andresen activator) exhibit an inflammatory reaction during condylar adaptation [34, 39]. This is a physiological reaction upon mandibular advancement and represents the preliminary stage to new bone formation. It appears reasonable, that this adaptation is slower in arthritis patients than in healthy subjects, since the “physiological” inflammation adds up on the antigen-induced TMJ arthritis. It might well be that bone modelling due to mandibular advancement was not completed at the time of sacrifice and thus the duration of the trial was possibly too short. This becomes particularly likely as none of the other parameters indicated a pathologic process (not more bone resorption, not more joint destruction). Furthermore it has to be remarked that although the AS animals did have a tendency to higher inflammatory markers than the A0 animals, this difference could not be statistically verified.

### Bone modelling

All rabbits showed a certain amount of bone modelling, which was expected, as all were growing animals. Whereas a more or less balanced amount of apposition and resorption was found for the untreated control group (00), the A0 animals exhibited a negative bone turnover (bone resorption > bone apposition). This bony resorption is most likely due to the induced arthritis and can often be verified in JIA-children [12, 13, 40, 41] where condylar erosions or flattening are found in MRIs and in extreme cases, even in orthopantomograms or ultrasound images [42, 43]. Compared to the A0 group, AS animals had significantly less bone resorption and in one case even more apposition than resorption. This positive effect is most likely due to the functional mandibular advancement. Although it is commonly known that functional therapy can induce bone apposition in the TMJ, this has so far not been evaluated in JIA patients. Recently Stoustrup et al. [21] described a positive effect of distraction appliances on JIA-caused facial asymmetries. Whether or not this positive effect is due to a skeletal reaction in the TMJ or due to a muscular adaptation can not be verified in a clinical study. The present data, however, suggest that a positive skeletal reaction can be obtained by functional orthopedic treatment even in cases with TMJ arthritis.

It was striking that if degenerative condylar changes occurred, they were always located in the anterior cranial region of the condyle, whereas the posterior areas were unaffected, indicating that mandibular growth was not restrained in a balanced way. Consequently the posterior condylar growth would continue normally, whereas the anterior-cranial growth direction would be restrained. This could lead to a posterior mandibular rotation (counter clockwise), resulting in an opening of the mandibular plane angle, as already described by Björk and Skieller [44, 45] in a case with an untreated JIA.

Limiting it has to be remarked that the results of this research can only partly be transferred to the clinical treatment of JIA children. Primarily it has to be considered that in this study an acute and early TMJ arthritis situation was analyzed, since the appliances were placed 1 week after the ovalbumin injection. It might well be that the amount of bone destruction caused in the TMJ during this 1 week would have been less if the protrusion appliance would have been inserted directly after the ovalbumin injection. However, in the clinical situation most JIA children that are being treated with functional appliances have already had an inflammation in the TMJ. Thus the fact that the appliances were placed 1 week after the TMJ arthritis has caused some degree of damage is closer to clinical reality. With the present study design, however, it remains unclear whether or not the mandibular protrusion has any preventive effects during inflammatory flares. Furthermore, the rabbits did not receive any kind of systemic medication, which would normally be a cofactor in the clinical situation in JIA children. In JIA patient treatment a large variety of drugs is applied (NSARs, glucocorticoids, DMARDs, biologica) [46, 47], which all can influence the course of the disease and whose positive or negative interactions with functional mandibular advancement are completely unknown. This should be evaluated in a further study. Additionally, in the present study only one single ovalbumin injection was performed in the TMJ which simulates the acute arthritis attack. This acute stage, however, was not reactivated through further reinjections and of course this might have had an impact on the findings after 60 days. However, it also has to be remarked that JIA children seldom have an acute TMJ inflammation over such a long time period and that also here the acute stage often becomes chronic, but of course this all should be evaluated in further studies.

## Conclusions

Despite a higher inflammatory reaction in arthritic joints, the insertion of a protrusion appliance in rabbits with an antigen induced TMJ arthritis lead to more bone apposition than in functionally untreated animals. Thus, the null hypothesis had to be rejected. Due to the relative small sample size and the large interindividual range of reactions, statistical significances could not be verified for all parameters. Since this study was performed on rabbits, the results can only be trend-setting as their transferability to JIA children is limited.

## Abbreviations

JIA: Juvenile idiopathic arthritis
TMJ: Temporomandibular joint

## Experimental groups:

AO: TMJ arthritis, no mandibular protrusion splint
AS: TMJ arthritis, mandibular protrusion splint
OO: no TMJ arthritis, no mandibular protrusion splint
OS: no TMJ arthritis, mandibular protrusion splint
